# Comparing Protein Stability in Modern and Ancient Sabkha Environments: Implications for Molecular Remnants on Ancient Mars

**DOI:** 10.3390/ijms26135978

**Published:** 2025-06-21

**Authors:** Qitao Hu, Ting Huang, Aili Zhu, Angélica Anglés, Osman Abdelghany, Alaa Ahmed, David C. Fernández-Remolar

**Affiliations:** 1 State Key Laboratory of Lunar and Planetary Sciences, Macau University of Science and Technology, Macau 999078, China; 2009853gse30001@student.must.edu.mo (Q.H.); thuang@must.edu.mo (T.H.); 2230024748@student.must.edu.mo (A.Z.); 2 CNSA Macau Center for Space Exploration and Science, Macau 999078, China; 3 Blue Marble Space Institute of Science, Seattle, WA 98104, USA; angelica.angles@bmsis.org; 4 Geosciences Department, College of Sciences, United Arab Emirates University, Al Ain 15551, United Arab Emirates; osman.abdelghany@uaeu.ac.ae (O.A.); aahmed83@uaeu.ac.ae (A.A.); 5 ISA, Iceland Space Agency, 101 Reykjavik, Iceland

**Keywords:** paleoproteomics, sabkha deposits, protein preservation, hypersaline environments, molecular biosignatures, Mars analogs, archaeal proteomics

## Abstract

Understanding the mechanisms of protein preservation in extreme environments is essential for identifying potential molecular biosignatures on Mars. In this study, we investigated five sabkha sedimentary samples from the Abu Dhabi coast, spanning from the present day to ~11,000 years before present (BP), to assess how mineralogy and environmental conditions influence long-term protein stability. Using LC-MS/MS and direct Data-independent Acquisition (DIA) proteomic analysis, we identified 722 protein groups and 1300 peptides, revealing a strong correlation between preservation and matrix composition. Carbonate- and silica-rich samples favored the retention of DNA-binding and metal-coordinating proteins via mineral–protein interactions, while halite- and gypsum-dominated facies showed lower recovery due to extreme salinity and reduced biomass input. Functional profiling revealed a shift from metabolic dominance in modern samples to genome maintenance strategies in ancient ones, indicating microbial adaptation to prolonged environmental stress. Contrary to expectations, some ancient samples preserved large, multi-domain proteins, suggesting that early mineral encapsulation can stabilize structurally complex biomolecules over millennial timescales. Taxonomic reconstruction based on preserved proteins showed broad archaeal diversity, including Thaumarchaeota and thermophilic lineages, expanding our understanding of microbial ecology in hypersaline systems. These findings highlight sabkhas as valuable analogs for Martian evaporitic environments and suggest that carbonate–silica matrices on Mars may offer optimal conditions for preserving ancient molecular traces of life.

## 1. Introduction

As a terrestrial planet with many similarities to Earth, Mars has long been a central focus in the exploration of planetary evolution and the search for life beyond our planet [1,2]. One of the most compelling lines of inquiry is the investigation of past, and potentially present, traces of life on Mars. Increasing evidence indicates the presence of liquid water on early Mars. High-resolution orbital imagery has identified extensive river valley networks and deltaic sedimentary formations, providing strong evidence that ancient watercourses traversed the Martian surface [3]. In situ exploration by the Curiosity rover at Gale Crater has shown that Mount Sharp, the central peak within the crater, is composed of layered sedimentary deposits representing a range of paleoenvironmental conditions [4]. Similarly, data from the ZhuRong rover have identified petrological features indicative of marine sedimentary rocks on the Martian surface [5]. These rocks display distinct bedding structures analogous to those found in coastal and shallow marine environments on Earth, including signs of bidirectional water flow—an indicator typically associated with tidal or marine settings [6]. Furthermore, numerous planetary missions have documented the widespread presence of salt deposits, especially chlorides and sulfates, across various Martian terrains [7], which suggest episodes of hypersaline conditions in the planet’s past [8,9].

With the evolution of Mars, extensive evaporation of water and low volume water input have caused most areas of Mars to become increasingly arid [10,11,12]. In this context, Earth’s sabkha environments serve as valuable analogues for investigating potential life and surface processes on Mars. Sabkhas—commonly found in arid coastal and inland basin regions—are typically flat or gently sloping low-lying areas subject to episodic flooding, evaporation, and sedimentation. A prominent example is the sabkha of the Arabian Peninsula, a vast flat terrain shaped by tidal influences and shallow groundwater processes. This topography favors the accumulation of fine sediments and evaporite minerals, such as halite, gypsum, and anhydrite [13,14]. Similarly, the Chott el Djerid in southern Tunisia represents an extensive sabkha system where evaporite deposits form through the evaporation of seawater or saline groundwater. As the sediments dry and contract, characteristic surface features, such as desiccation cracks and polygonal patterns, emerge that are features also observed on Mars [15]. The extreme conditions in sabkhas, with high salinity, fluctuating water activity, and temperature extremes, mimic the current surface environment of Mars. Salts, in particular, have the ability to stabilize liquid water under low-temperature and low-pressure conditions [16]. Consequently, high-salinity regions on Mars that resemble sabkha systems may be key targets for preserving biosignatures and potential traces of ancient life.

For studying the preservation of biological macromolecules in Mars’ hypersaline conditions, proteins rather than DNA possess more complex multi-dimensional structures, making them more resistant to degradation compared to DNA [17]. Amino acids are linked by peptide bonds and form a variety of secondary and tertiary structures, enabling proteins to form relatively stable conformations under certain circumstances [18,19]. In the hypersaline environment of the sabkha, the solubility and charge state of proteins may be significantly influenced by the high salt concentrations. However, many proteins exhibit remarkable adaptability to these conditions by adjusting their structural conformation. These adaptive mechanisms can involve modifications such as changes in the surface charge distribution, folding patterns, or forming more stable interactions among amino acids to maintain function and integrity [20]. Such structural flexibility allows proteins to retain their biological activity even in extreme environments, highlighting their resilience and adaptability to osmotic stress.

In this regard, halophilic microorganisms, widely distributed in saline environments across the globe, are a group that survives under salinity conditions ranging from 1% to 30%. Their salt tolerance mechanisms beside the protein structure modification include, but are not limited to, having relatively thick cell walls [21]. These walls contain various lipoproteins, and acidic proteins such as glycoproteins can form negatively charged regions to attract Na^+^ in hypersaline environments. Part of the cell membranes of halophilic bacteria are distributed with membrane purple pigments containing rhodopsin, which can promote the transfer of H^+^ by absorbing visible light, generating a potential difference across the plasma membrane and thereby synthesizing Adenosine Triphosphate (ATP) to store the energy required for their vital activities. The ion concentration within the intracellular matrix is comparable to that of the external environment to resist dehydration caused by the extracellular hypersaline environment. The intracellular K^+^ concentration is as high as 7 mol/L, far higher than that outside the cell, while the situation of Na^+^ concentration is the opposite, with a lower concentration inside and a higher concentration outside, endowing halophilic bacteria with sufficient sodium-excreting and potassium-absorbing capabilities [22]. Consequently, these halophile proteins adapted to high saline concentration inherently possess the property of being less prone to decomposition in sabkha, which is conducive to our exploration of past life traces in the hypersaline environment of Mars.

In this research, we investigate several ancient sabkha materials outcropping along the coast of Abu Dhabi, United Arab Emirates, to examine the early stages of protein preservation during the Holocene. We have selected five representative sedimentary materials with varying mineral composition, biological content, and age formed since the early Holocene. By comparing the types and characteristics of these samples and analyzing the mass spectrometry results of their proteins, we aim to better understand the process of protein preservation in early-stage hypersaline environments. This research may provide insights into the potential preservation of complex molecular biosignatures in the hypersaline regions of Mars.

## 2. Results and Discussion

### 2.1. Age and Mineralogical Characteristics

Radiocarbon dating results revealed distinct age populations for the sabkha deposits (Table 1). Sample D yielded the oldest age range of 11.0 ± 0.2 ka BP, representing early Holocene deposits, while sample B dated to 2.7 ± 0.2 ka BP, corresponding to Late Holocene deposition. Samples A and E represent modern sabkha deposits, whereas sample C, although not directly dated, is considered contemporaneous with Sample B based on their shared stratigraphic position. Despite their temporal equivalence, Samples B and C exhibit distinct lithological characteristics, as discussed below.

**Figure 1:**
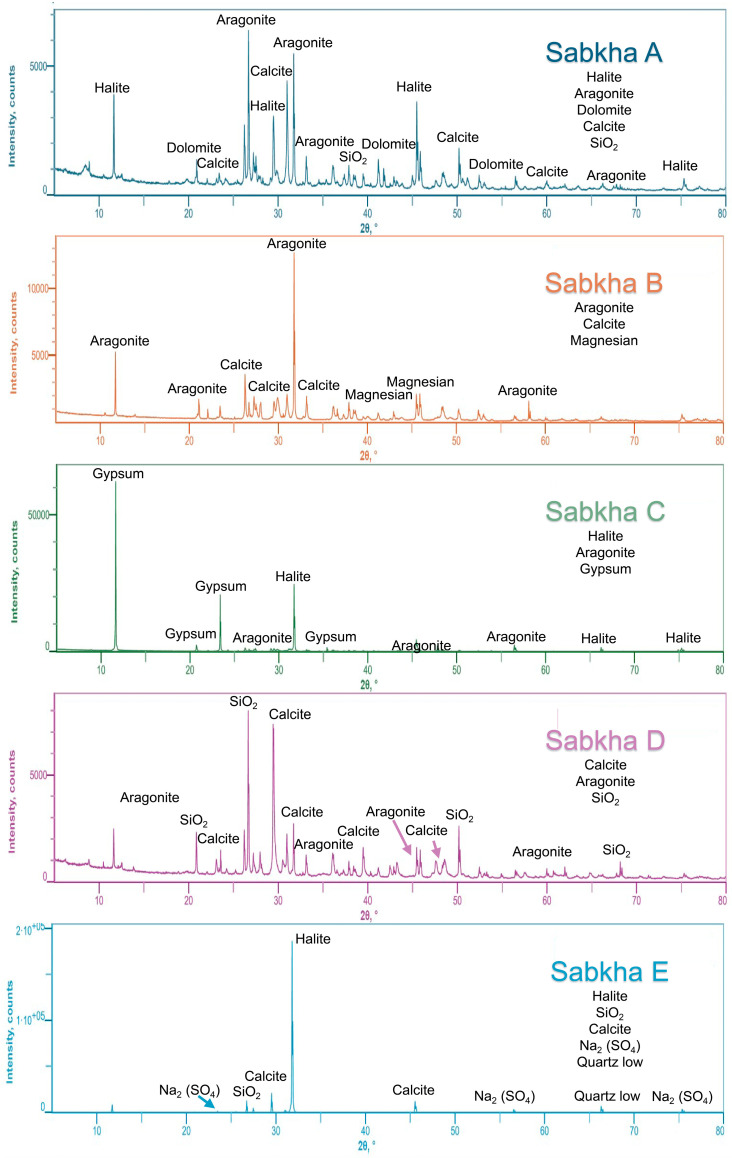
XRD patterns for sediment samples collected from five different sabkha environments, ranging from modern deposits to early Holocene formations, as detailed in Table 1.

X-ray diffraction (XRD) analysis of Samples A to E revealed a complex mineral assemblage (Figure 1) including halite (NaCl), aragonite (CaCO_3_), dolomite [CaMg(CO_3_)_2_], calcite (CaCO_3_), α-quartz (SiO_2_), magnesite (MgCO_3_), gypsum (CaSO_4_·2H_2_O), and thenardite (Na_2_SO_4_). All five samples exhibited characteristic diffraction peaks at 2θ angles of approximately 26°, 30°, 40°, and 45°.

Sample A (modern sabkha) displayed the most complex diffraction pattern with 93 distinct peaks, including significant reflections beyond 2θ = 50°. The primary mineral assemblage comprises halite (NaCl), aragonite (CaCO_3_), dolomite [CaMg(CO_3_)_2_], calcite (CaCO_3_), and α-quartz (SiO_2_). The presence of aragonite may indicate secondary mineralization under specific diagenetic conditions.

Sample E (modern sabkha) exhibited a prominent diffraction peak at 2θ = 31.78°, consistent with the observed halite-encrusted surface. The mineralogical composition is dominated by halite (NaCl), with subordinate α-quartz (SiO_2_), calcite (CaCO_3_), thenardite (Na_2_SO_4_), and low-temperature α-quartz. The presence of thenardite likely represents secondary mineralization through evaporative concentration and chemical precipitation in the sabkha environment.

Samples B and C, collected from the younger sabkha terrace (T1), show distinct mineralogical compositions. Sample B exhibited multiple diffraction peaks but a relatively simple mineralogy, dominated by aragonite (CaCO_3_), calcite (CaCO_3_), and minor magnesian calcite [(Mg,Ca)CO_3_)], reflecting a predominantly carbonate assemblage. Sample C showed fewer diffraction peaks but a more diverse mineral assemblage compared to Sample B, including prominent halite (NaCl) and aragonite (CaCO_3_). The presence of gypsum (CaSO_4_·2H_2_O), identified across multiple diffraction peaks, likely represents secondary mineralization through evaporative concentration and chemical precipitation.

Sample D, representing the oldest sabkha deposits collected (ca. 11,000 years BP), exhibits a mineral assemblage dominated by calcite (CaCO_3_), aragonite (CaCO_3_), and α-quartz (SiO_2_), with a notably low presence of chloride salts. Rather than resulting solely from post-depositional diagenetic alteration, this mineralogical composition is best interpreted as a reflection of the original paleoenvironmental conditions during the early Holocene. At that time, regional climate patterns were characterized by reduced salinity and increased humidity, consistent with the onset of the African Humid Period and broader post-glacial climatic amelioration [23,24].

The dominance of carbonate minerals, especially in early sabkha deposits, suggests precipitation under lower salinity regimes, where the balance between evaporation and groundwater input favored carbonate saturation over chloride crystallization [25,26]. Under such conditions, the accumulation of soluble salts, such as halite or other chlorides, would have been limited, not only due to lower evaporative concentration but also to enhanced groundwater flushing [27].

In this context, the scarcity of chloride salts in Sample D may be partially influenced by diagenetic leaching, but it more directly records primary depositional settings where hypersalinity was not yet dominant. The relatively stable mineral phases, such as calcite and aragonite, point to early carbonate precipitation, potentially assisted by microbial activity. Microbial mats, particularly cyanobacteria, could have promoted carbonate formation via photosynthetic uptake of CO_2_, leading to increased pH and carbonate supersaturation [28,29]. Furthermore, microbial biomineralization processes likely contributed to the formation of biogenic carbonates, which, upon the death and decay of microbial communities, became incorporated into the accumulating sabkha sediments [30].

The presence of α-quartz is consistent with aeolian or detrital input and may reflect the aridification trend that followed the initial humid phase, or sediment transport from surrounding highlands. Overall, the mineralogical profile of Sample D reflects a less saline, more hydrologically active sabkha environment, capturing the transition from wetter Early Holocene conditions to increasingly evaporative dynamics that later characterized the sabkha system. The predominance of carbonate minerals and relative scarcity of chloride salts in Sample D may have significant implications for biomolecule preservation. The more stable carbonate-dominated matrix potentially provides better conditions for long-term preservation of organic materials, while the reduction in chloride salts through diagenetic processes may have created favorable conditions for biomolecule retention within the sediment.

### 2.2. Protein Concentration and Distribution

Qubit 4 analysis of Samples A to E yielded mean total protein concentrations (TPC, including proteins and peptides) of 540 ng⋅μL^−1^, 438 ng⋅μL^−1^, 452 ng⋅μL^−1^, 494 ng⋅μL^−1^, and 332 ng⋅μL^−1^, respectively (Table 1), which were sufficient for subsequent LC-MS analysis. The TPC patterns across samples revealed unexpected trends. Sample A, containing diverse mineral phases and microbial mat material, yielded a protein concentration only marginally higher than Sample E, which showed the lowest concentration. This observation may be explained by the exposure of Sample A’s microbial mat to surface evaporation, leading to desiccation. Protein and peptide hydrolysis generally occurs rapidly in organisms that are not quickly buried after death. However, the formation of a desiccated protective outer layer, together with the presence of abundant halite (NaCl), appears to have significantly slowed this degradation process, facilitating the partial preservation of proteins. Sample E, a modern halite precipitate formed from sabkha brines, likely contains proteins produced by halophilic microorganisms adapted to hypersaline conditions, rather than preserved ancient biomolecules. This suggests active microbial communities inhabiting the brines at the time of salt formation, providing insights into the ecological dynamics of modern evaporitic environments.

Samples B and C, collected from the younger terrace, exhibited similar TPC despite their distinct mineralogical compositions, suggesting effective preservation over millennial timescales. Unexpectedly, Sample D, representing the oldest deposits, showed relatively high TPC. This sample differs from Samples B and C in its mineralized character, being dominated by carbonate minerals (CaCO_3_, CaMg(CO_3_)_2_), with subordinate amounts of sulfates, chlorides, and silicates (SiO_2_). The preservation of proteins through complex diagenetic processes suggests that carbonate-rich sabkha facies may offer favorable conditions for long-term biomolecule stability. While a contribution from living halophilic microbial communities within the sediments cannot be excluded, it is noteworthy that Samples B and C—although also ancient (2918–2502 cal BP)—exhibit lower protein concentrations compared to Sample D (11,231–10,770 cal BP). This difference may be related not only to the higher carbonate content in Sample D, but also to the less extreme (i.e., less hypersaline) conditions during its formation, which could have supported greater microbial diversity and biomass input, thereby enhancing the initial protein pool available for preservation.

**Figure 2:**
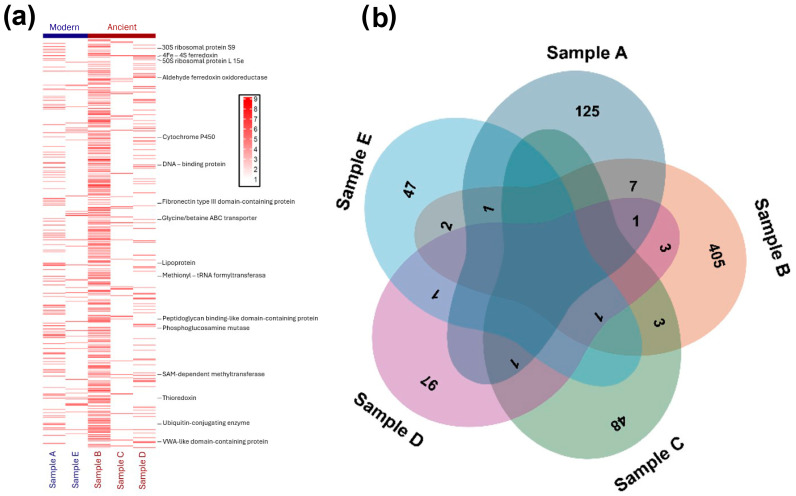
Proteomic analysis of sabkha deposits. (**a**) Heatmap displaying the coverage of various proteins across different Samples A to E. The intensity of red shading represents the logarithmically transformed coverage values (1–9), where darker red indicates higher coverage. The numerical scale (right) visually enhances the band patterns for easier comparison. Samples are grouped into “Ancient” (dark red) and “Modern” (blue) categories, as indicated in the top bar. (**b**) Venn diagram displaying the quantitative distribution and overlap of identified proteins among the five samples. Numbers indicate unique and shared proteins between sample sets.

Mass spectrometry analysis using MaxQuant revealed the identification of 1300 peptides and 722 protein groups across all samples (Figure 2b). The modern deposits yielded 125 proteins from Sample A and 47 proteins from Sample E. Among the ancient deposits, Sample B contained the highest number of proteins (405), while Samples C and D yielded 48 and 97 proteins, respectively. This disparity between peptide and protein counts is characteristic of ancient geological samples, reflecting extensive protein degradation over time.

The relationship between protein concentrations and mass spectrometry results revealed notable inconsistencies. While total protein concentration (TPC) provides a general estimate of the peptidic biomass present in each sample—serving as a proxy for the initial biological input—the number of proteins and peptides identified, and particularly their ratio, offers further insight into the degradation state of the protein pool. A high peptide count relative to proteins typically indicates more advanced hydrolysis, while a higher protein-to-peptide ratio suggests better preservation of intact proteins.

Samples A and D, despite showing the highest TPC values (540 and 494, respectively), did not yield correspondingly high numbers of intact proteins. These discrepancies likely arise from different preservation mechanisms. Sample A’s complex depositional environment may have limited protein diversity preservation, while Sample D’s lower protein count, despite high TPC, likely reflects time-dependent degradation processes. The latter may have resulted in the persistence of lower-order protein structures and peptide fragments, consistent with an aged protein pool subjected to prolonged diagenetic alteration.

Samples B and C exhibit similar TPC values (438 and 452), suggesting comparable total peptidic inputs. However, they differ markedly in the number of proteins identified—405 in Sample B versus only 48 in Sample C—and in their protein-to-peptide ratios (0.75 vs. 0.29). While random sampling effects cannot be ruled out, this disparity likely reflects differences in mineralogical composition and depositional context. Sample B, with a carbonate-rich matrix (aragonite, calcite, and Mg-calcite) and moderate salinity (60–90 PSU), appears to offer more favorable conditions for protein preservation, as indicated by its high protein recovery and minimal degradation. In contrast, Sample C, dominated by gypsum and halite and deposited under more extreme hypersaline conditions (100–150 PSU), shows both a lower protein-to-peptide ratio and reduced protein count. This may result not only from enhanced degradation but also from reduced microbial biomass input due to salinity stress at the time of deposition.

Sample E also shows limited protein recovery (47 proteins) despite being a modern halite precipitate. Its very high estimated salinity (300–350 PSU) and exposed setting likely inhibited microbial activity, leading to low initial biomass input. Its low protein-to-peptide ratio (0.34) may further reflect abiotic hydrolysis under extreme saline stress, although biological input appears to be the primary limiting factor.

The preservation of proteins and peptides in sedimentary environments is thus influenced by multiple interacting factors, including age, mineral composition, salinity, and water activity. Protein degradation breaks down large molecules into peptide fragments, but this does not always result in a straightforward increase in peptide abundance due to secondary processes like adsorption, hydrolysis, or microbial reworking. Sample B (2918–2502 cal BP) stands out as the most favorable in terms of both preservation and peptide stability, with high protein (405) and peptide (539) counts and the highest protein-to-peptide ratio (0.75). Its carbonate matrix likely promoted protein adsorption and limited degradation. By contrast, Sample C, despite being of similar age, shows drastically lower values for both proteins and peptides, likely due to the chemically aggressive nature of its gypsum/halite matrix, compounded by lower biological input.

For the oldest sample, Sample D (~11,000 years), the relatively high TPC (494) suggests a larger original biomass contribution, likely due to less extreme salinity and more favorable ecological conditions at the time of deposition. However, the moderate salinity and water activity in this sample may have also allowed greater microbial and enzymatic activity over time, promoting progressive degradation. The result is a modest protein count (97) and a lower protein-to-peptide ratio (0.46), reflecting significant diagenetic alteration of an initially richer protein pool.

Together, these observations underscore that TPC reflects the quantity of peptidic biomass initially deposited, while the ratio of proteins to peptides serves as a useful indicator of the degree of degradation in the sedimentary protein pool. Variations across samples highlight the role of mineral composition, salinity, and depositional environment in controlling both the preservation and detectability of proteins and peptides in sabkha systems.

The interplay between degradation and preservation mechanisms in hypersaline environments presents a nuanced picture of protein taphonomy. In the most saline samples (C and E), we observed a complex balance of opposing processes. On the one hand, extreme hypersalinity ( > 100 PSU) appears to enhance protein hydrolysis through increased ionic strength and reduced protein stability, evidenced by the lower protein-to-peptide ratios (0.29 and 0.34, respectively). This suggests that beyond certain salinity thresholds, the denaturing effects of salt may outweigh its preservative qualities [31]. Conversely, the halite-rich matrices in these samples likely contributed to preservation through water activity reduction and desiccation, which limited enzymatic and microbial degradation, consistent with findings by Georgiou et al. (2015) [32]. Such preservation effects, however, were counterbalanced by the aggressive chemical environment of gypsum/halite matrices, which may catalyze certain hydrolytic reactions as demonstrated by Stan-Lotter and Fendrihan (2015) [33]. Additionally, the extreme salinities in these samples appear to have constrained the initial biological input by limiting microbial diversity and biomass, resulting in smaller original protein pools available for preservation [34]. These findings suggest that, while moderate hypersalinity (as in Sample B) offers optimal conditions for protein preservation through both limited biological activity and chemical stabilization, more extreme hypersaline conditions introduce additional degradative pathways that can accelerate protein hydrolysis despite concurrent preservation mechanisms [35]. This delicate balance of preservation versus degradation in hypersaline environments has significant implications for our understanding of protein diagenesis in evaporitic settings and for the search for ancient biomolecules in analogous extreme environments on Earth and potentially beyond.

### 2.3. Protein Identification and Classification

The protein analysis of sabkha samples yielded two complementary dimensions of information. First, it provided insight into preservation mechanisms by identifying which protein structures and classes persist over geological timescales in hypersaline environments. Second, it offered detailed taxonomic characterization of the microbial communities that originally produced these biomolecules. This dual approach allowed us to simultaneously investigate both taphonomic processes affecting molecular preservation and reconstruct the microbial ecology of these environments through time.

The mass spectrometry and bioinformatic pipeline we employed not only quantified total protein concentrations and identified specific preserved proteins but also enabled phylogenetic classification of these proteins to their source organisms. This taxonomic resolution revealed unexpected diversity within the archaeal communities, including lineages not typically associated with hypersaline environments. By integrating preservation patterns with taxonomic distributions, we gain a more comprehensive understanding of how environmental conditions, microbial community structure, and molecular taphonomy interact in these Mars-analog settings.

#### 2.3.1. Classification of Preserved Proteins

Upon obtaining proteomic data from sabkha samples, we first conducted a statistical analysis of protein group coverage values for each sample. The initially identified proteins were then mapped to UniProt accession IDs using the UniProt ID mapping tool to create a standardized and functionally annotated dataset of sabkha-associated proteins (Supplementary Materials S2). After log-transforming the coverage values, a heatmap was generated to visualize protein abundance across samples (Figure 2a). Proteins linked to long-term preservation in sabkha environments were annotated and classified into seven major categories: (1) small-molecular-weight proteins capable of embedding into mineral pores (e.g., small ribosomal proteins and thioredoxin < 12 kDa) [36]; (2) environmentally sensitive proteins (e.g., methionyl-tRNA formyl transferase, non-specific serine/threonine protein kinase) [37]; (3) structurally stable proteins [38,39], including domain-containing and structural proteins (e.g., glycine/betaine ABC transporter, DNA-binding protein, fibronectin type III domain-containing protein); (4) crosslinking-capable proteins [40], such as disulfide-bonded or enzymatically crosslinked proteins (e.g., SAM-dependent methyltransferase, peptidoglycan binding-like domain-containing protein); (5) metal-binding proteins central to core metabolic pathways (e.g., 4Fe-4S dicluster domain-containing protein, 4Fe-4S ferredoxin, cytochrome P450, zinc metalloprotease, nitrogenase protein alpha chain) [41]; (6) degradation-resistant modified proteins [42], including glycosyltransferase, phosphoglucosamine mutase, SAM-dependent methyltransferase, and ubiquitin-conjugating enzyme; (7) amino acid-enriched proteins interacting with mineral cations, such as acidic residue-rich proteins (e.g., aldehyde ferredoxin oxidoreductase, and VWA-like domain-containing protein) [43] and hydrophobic residue-rich proteins (e.g., lipoprotein, membrane protease YdiL) [44].

The extreme geochemical conditions of sabkha environments (hypersalinity, evaporitic mineral precipitation, redox fluctuations) regulate protein preservation through multi-scale interactions. Short-term preservation in modern samples (A and E) is dominated by physical encapsulation mechanisms. For example, the high abundance of lipoprotein (hydrophobic membrane-anchored protein; coverage: 4.93) and methionyl-tRNA formyltransferase (coverage: 5.19) in modern Sample A reflects rapid gypsum/salt crystallization-mediated protection of membrane proteins and oxidation-sensitive enzymes [39]. In contrast, proteins in the ancient samples (B–D) appear to be stabilized through chemical mechanisms. Metal-binding proteins—such as 4Fe–4S ferredoxins and zinc metalloproteases—likely gain structural rigidity via Fe^2+^ and Zn^2+^ coordination, potentially interacting with mineral surface cations (e.g., coverage in Sample D: 6.19–5.17). Additionally, post-translational modifications such as methylation (mediated by SAM-dependent methyltransferases) and glycosylation (via glycosyltransferases) may further protect proteins by reducing hydrophilicity and thereby limiting hydrolytic degradation [45].

A particularly striking finding from the ancient sedimentary profile is the exclusive presence of archaeal DNA-binding proteins (coverage: 4.91) in the oldest materials (Sample D). These proteins play a central role in the survival strategies of extremotolerant archaea, contributing to genome stabilization, transcriptional regulation, and chromatin compaction through structural reorganization [46]. One well-known example is the Sta1 protein from Thermococcus kodakarensis, which uses ATP to remodel chromatin and fine-tune transcription by altering DNA topology [47]. Among these, Dps-like proteins stand out as a key adaptation. These proteins serve dual functions: they sequester iron and mitigate oxidative stress, thereby reducing hydroxyl radical generation through Fenton chemistry. At the same time, they compact DNA, helping to maintain genomic integrity under redox-imbalanced and oxidizing conditions [48].

The DNA-binding protein exclusively detected in the oldest sample, Sample D, can be attributed to the synergistic effect of silica and calcite minerals. This silica–calcite matrix appears crucial for long-term preservation of DNA-binding proteins specifically, despite Sample B containing more total proteins and carbonates. Similar preservation phenomena have been documented by Alleon et al. (2016) [41], who demonstrated that silica-rich environments create protective microenvironments that shield biomolecules from degradation through surface adsorption and reduced water activity. The preferential preservation of DNA-binding proteins in siliceous sediments has been further supported by Briggs and McMahon (2016) [44], who found that silica can rapidly entomb proteins and create mineral replacements that preserve molecular information. Additionally, Ana Robles-Fernández et al. (2022) [49] observed that calcite in combination with silica establishes favorable pH microenvironments that specifically enhance preservation of proteins with high isoelectric points, a characteristic of many DNA-binding proteins. This differential preservation highlights that biomolecular taphonomy in hypersaline environments is governed more by specific mineral associations than by chronological age, with the silica–calcite matrix in Sample D providing an optimal preservation environment for DNA-binding proteins despite its considerable antiquity.

Protein preservation modes exhibit time-dependent shifts from modern to ancient samples. Initial-stage preservation is strongly influenced by reductive microenvironments and low water activity, as evidenced by elevated thioredoxin abundance in the modern samples, A and E (coverage: 4.86–4.64). Its near-complete absence in ancient samples (coverage approaching zero) reflects degradation driven by oxidative porewater conditions during early sabkha diagenesis [50]. Long-term protein preservation is governed by progressive mineral–protein chemical interactions. The presence of the nitrogenase alpha chain (Fe-Mo cofactor protein; coverage: 4.40 in Sample C) indicates that localized anoxic microenvironments persisted within the evaporitic matrix, supporting continued stabilization beyond initial diagenesis. Likewise, the persistence of phosphoglucosamine mutase (coverage: 6.38 in Sample B) reflects direct stabilization through interactions with Mg^2+^ ions in the magnesium calcite matrix [51]. In the oldest sample, D (11,231–10,770 cal BP), selective preservation of DNA-binding proteins (coverage: 4.91) within a calcite–silica matrix points to protective effects from silica through surface adsorption and reduced water activity during early mineral entrapment [41]. This spatial heterogeneity underscores diagenetic controls where mineralogical composition, rather than age alone, exerts a dominant influence—calcite–silica matrices preferentially preserve positively charged proteins, while evaporitic minerals (gypsum/halite) favor the retention of metal-binding proteins (e.g., zinc metalloprotease, coverage: 5.17) and proteins enriched in acidic residues (e.g., aldehyde ferredoxin oxidoreductase, coverage: 5.68).

These findings underscore that protein preservation is both time- and environment-dependent, with early diagenetic loss of redox-sensitive proteins contrasted by the long-term survival of others through mineral-mediated stabilization. This selective retention highlights a potential bias in the protein fossil record, where absence does not necessarily indicate original absence, but rather reflects differential preservation. Recognizing these pathways is essential when interpreting ancient biosignatures, particularly in analog systems where similar preservation dynamics may apply to early Earth or Martian environments.

**Figure 3:**
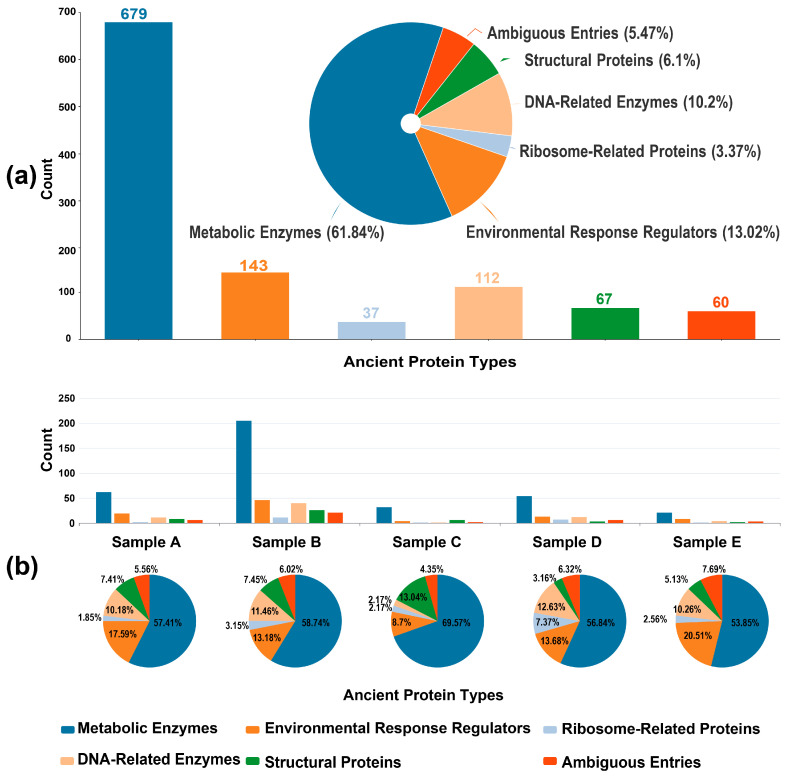
(**a**) Functional classification of 1098 non-redundant proteins identified across all sabkha samples after UniProt-based annotation. Proteins were grouped into six major categories: Metabolic Enzymes (61.84%), Environmental Response Regulators (13.02%), DNA-Related Enzymes (10.20%), Structural Proteins (6.10%), Ribosome-Related Proteins (3.37%), and Ambiguous Entries (5.47%) comprising multifunctional or poorly characterized proteins. The absolute count of proteins per category is shown via bar chart, while the proportional distribution is represented by the central pie chart. (**b**) Sample-specific breakdown of functional protein classes for Samples A–E. Bar charts indicate total counts per protein class, while accompanying pie charts display their relative proportions within each sample. All samples are dominated by metabolic enzymes and environmental response regulators, with notable increases in DNA-related enzymes and ribosomal proteins in older samples (e.g., Sample D), reflecting functional shifts associated with long-term microbial survival strategies under hypersaline and nutrient-limited conditions.

A total of 1098 non-redundant proteins were identified across the sabkha system following proteomic analysis and UniProt-based annotation (Supplementary Materials S2). After removing uncharacterized entries, domains of unknown function (DUFs), and redundant sequences, proteins were classified into five major functional categories (Figure 3a): metabolic enzymes (61.84%), environmental response and regulatory proteins (13.02%), ribosomal proteins (3.37%), DNA-associated enzymes (10.20%), and structural proteins (6.10%). An additional 5.47% could not be definitively assigned due to multifunctional annotations. This functional profile is broadly consistent with microbial energy metabolism and environmental adaptation in hypersaline ecosystems [34], where the enrichment of ABC transporters and histidine kinases within the environmental response group reflects known osmoregulatory and signal transduction mechanisms in extreme halophilic archaea [52].

Comparative analysis across five samples, spanning modern to early Holocene deposits, revealed distinct patterns in protein functional group distributions (Figure 3b). All samples shared a dominant presence of metabolic enzymes (53–70%) and environmental response proteins (8–21%), including glycolytic enzymes and compatible solute synthases—key components of osmoadaptation strategies [53]. Sample B exhibited elevated levels of DNA-associated enzymes (18%) and ribosomal proteins (6%), potentially reflecting microbial proliferation driven by episodic freshwater influx [54]. In contrast, Sample C, with a similar age (Table 1), presented reduced metabolic diversity but a marked increase in structural proteins (13.04%), suggesting adaptation to prolonged aridity through reinforcement of cell envelope structures [55].

Compared to Samples B and C, the oldest sample, D (Table 1), displayed elevated proportions of DNA-associated enzymes (12.63%) and ribosomal proteins (7.37%), while structural proteins were comparatively underrepresented (3.16%). We interpret this shift as a functional signature of prolonged environmental stress associated with extended burial time and diagenesis. Under conditions of sustained nutrient limitation and high salinity, microbial communities likely entered dormant or metabolically quiescent states, conserving energy by maintaining proteins essential for genomic stability and basic cellular readiness. This includes the preferential retention of DNA repair enzymes (e.g., RecA, DNA polymerases) and ribosomal proteins, which support genome maintenance and enable rapid translational reactivation upon environmental improvement, while synthesis of energetically costly structural proteins (e.g., cytoskeletal and membrane-associated proteins) is suppressed [56]. This functional strategy is consistent with known responses in halophilic archaea, which accumulate compatible solutes and upregulate DNA repair pathways while downregulating genes involved in cell wall synthesis under prolonged hypersaline stress [57]. Previous research studies have further illuminated this adaptive mechanism, demonstrating the systematic genetic and protein expression adjustments microorganisms employ to survive extreme conditions [21]. In the same period, studies on the different osmotic adaptation strategies of strictly halophilic bacteria and halotolerant bacteria isolated from the northwestern Himalayas have shown a profound metabolic shift towards genome maintenance and stress tolerance, characterized by increased expression of stress-responsive proteins including RecA, Dps-like proteins, and ATP-dependent chaperones [22]. These observations suggest a shift toward a “survival over growth” adaptive state in the archaeal component of sabkha microbial communities during late-stage sedimentation, demonstrating the remarkable ability of microorganisms to dynamically reconfigure their cellular machinery in response to persistent environmental challenges.

From a paleoecological perspective, the sabkha microbial communities appear to have expressed stress-response strategies characterized by a shift from “metabolic dominance” to enhanced “genome maintenance,” consistent with adaptation to hypersaline, nutrient-limited, and desiccating conditions near the sediment surface [58]. This trend is evidenced by the increased representation of DNA-associated enzymes and ribosomal proteins in older samples. However, these proteins were likely expressed during early environmental stress events rather than as a consequence of extended burial. Notably, Sample D is enriched in carbonates and silica, suggesting that rapid mineralization during sedimentation may have physically encapsulated microbial communities, effectively preserving their proteomic profile close to the time of entrapment. Such early mineral precipitation could have shielded redox-sensitive and stress-related proteins from degradation by limiting water activity and enzymatic turnover, thereby favoring the long-term retention of genome maintenance signatures in the sedimentary record. This interpretation is supported by experimental and natural observations of protein stabilization via silica adsorption and carbonate encapsulation under low-permeability conditions [41,59]. Approximately 12% of the protein dataset remains unclassified, including candidates for novel halotolerant proteins and ion channel components, offering promising avenues for investigating early osmoadaptive mechanisms. Future integration with nanoscale secondary ion mass spectrometry (NanoSIMS) could enable high-resolution correlation of metabolic patterns with paleoenvironmental variables, refining our understanding of microbial survival strategies in evaporitic systems.

#### 2.3.2. Length-Dependent Preservation Patterns in Ancient Proteomes

**Figure 4:**
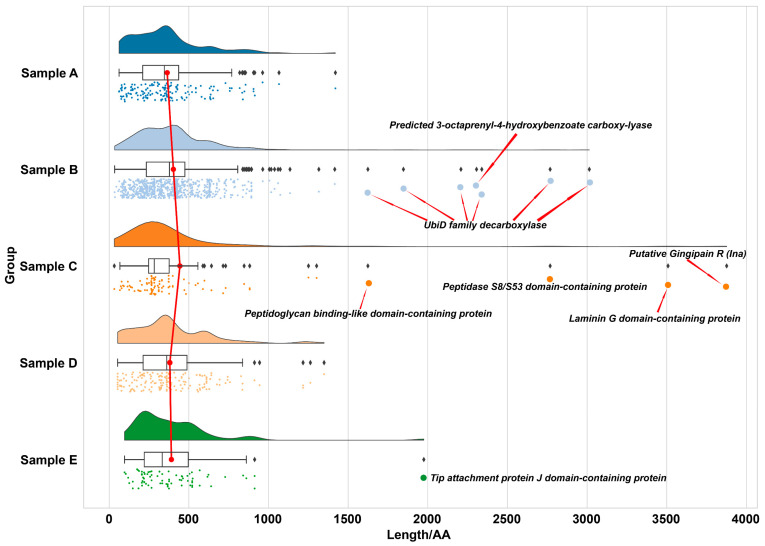
Length distribution of preserved proteins across sabkha samples. The x-axis denotes protein length in amino acids (AA), while the y-axis differentiates Samples A–E by stratigraphic position and age. Each dot represents an individual protein identified via LC-MS/MS, color-coded by sample. Violin plots and boxplots overlay the distributions, illustrating both density and quartile spread. A red line connects the median protein lengths of each sample to visualize central tendency trends across the sequence. Proteins exceeding 1500 AA are highlighted and annotated with representative domain-rich proteins, including Putative Gingipain R (Ina), Peptidoglycan-binding-like domain-containing protein, and UbiD family decarboxylase. Notably, intermediate-aged Samples B and C exhibit greater protein length variability and contain the highest number of long, multi-domain proteins. In contrast, both modern samples (A and E) and the oldest, Sample D, display predominantly shorter protein profiles. These patterns suggest that protein preservation is not linearly related to sample age but is strongly modulated by depositional environment, mineral matrix, and intrinsic protein features such as domain architecture.

Protein length distributions across sabkha samples, expressed in amino acid (AA) residues, were determined by mapping identified proteins to UniProt accession entries (Figure 4). The majority of preserved proteins fall below 1000 AA, with a strong concentration in the 300–600 AA range across all samples (Supplementary Materials S2). Modern samples (A and E) display a tighter clustering of shorter proteins, mostly within 100–500 AA, with only rare outliers extending beyond 1500 AA. In contrast, older samples, particularly Samples B and C, exhibit greater length variability and a higher incidence of large, multi-domain proteins exceeding 1500 AA.

Sample B is notably enriched in ubiquitin-like proteins, including UbiD family decarboxylase and predicted 3-octaprenyl-4-hydroxybenzoate carboxy-lyase—enzymes characterized by conserved β-grasp fold domains known to enhance structural integrity and resistance to unfolding [60]. Sample C preserves the longest protein identified in the dataset, a putative Gingipain R (Ina) spanning 3876 AA. This cysteine protease contains a canonical protease domain that contributes to enzymatic stability and substrate specificity, features commonly associated with resilience under harsh physicochemical conditions [61]. Other notable long proteins in Sample C include Laminin G and Peptidoglycan-binding-like domain-containing proteins, all consistent with complex, multi-domain architectures.

Despite its antiquity, Sample D lacks preserved proteins longer than 1500 AA, though it retains a significant population within the 500–1000 AA range. This may reflect rapid early mineral encapsulation in carbonate–silica matrices, which limited degradation but did not favor the stabilization of very large protein structures.

Contrary to earlier assumptions that protein length would inversely correlate with age—due to progressive degradation over time [62]—the dataset reveals a more nuanced pattern. Modern samples contain predominantly shorter proteins, while longer and structurally complex proteins appear in intermediate-age samples. These results underscore that protein preservation is not solely governed by chronological age, but rather by a combination of environmental factors (e.g., salinity, pH, redox conditions), mineralogical context, and intrinsic protein properties such as domain structure and degradation resistance. The combined proteomic and mineralogical data support a model in which rapid sediment entrapment and favorable mineral matrices are central to the long-term survival of larger and functionally complex proteins.

These results underscore a transition in preservation mechanisms across the sabkha system, moving from physical encapsulation in modern samples to chemical stabilization in ancient deposits. In recent evaporitic environments, rapid mineral precipitation appears to entomb proteins quickly, limiting their exposure to degradative agents. This is exemplified by the retention of environmentally sensitive enzymes such as methionyl-tRNA formyl transferase and membrane-associated lipoproteins.

In contrast, long-term preservation in older samples, such as Sample D, appears increasingly governed by molecular interactions between proteins and the mineral matrix. The preferential retention of metal-binding proteins and proteins with stable tertiary structures points to the importance of chemical stabilization via coordination with divalent cations (e.g., Fe^2+^, Zn^2+^), surface adsorption, and micro-environmental buffering. These interactions may form highly stable organo-mineral complexes capable of persisting despite diagenetic fluid alterations.

This shift has direct implications for the detection of ancient biosignatures on Mars, where preservation timescales are orders of magnitude longer. If similar mineralogical and geochemical mechanisms operate in Martian sediments, then the surviving biosignature record would likely be biased toward proteins or peptides with robust structural features, metal-binding domains, and favorable interactions with host minerals—rather than representing the original diversity of the Martian proteome.

#### 2.3.3. Taxonomic Characterization and Microbial Community Reconstruction Through Proteomics

**Figure 5:**
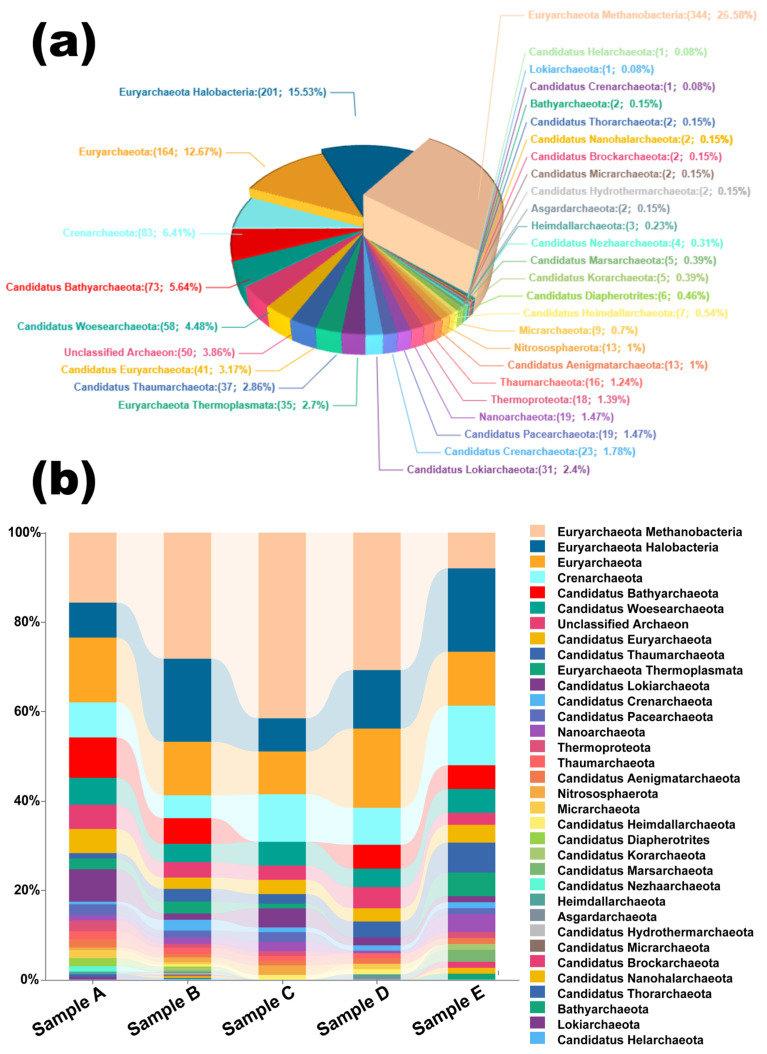
Taxonomic composition and distribution of archaeal proteins in sabkha samples. (**a**) Pie chart illustrating the overall archaeal community structure based on proteomic assignments across all five samples. Taxonomic classification was performed at the phylum level using UniProt ID mapping. Dominant lineages include Euryarchaeota Halobacteria (15.53%), Euryarchaeota (12.67%), Crenarchaeota (6.41%), Candidatus Bathyarchaeota (5.64%), and Candidatus Woesearchaeota (4.48%). Minor yet ecologically intriguing groups include Candidatus Thaumarchaeota, Thermoplasmata, and several deep-branching candidate phyla such as Lokiarchaeota, Nezhaarchaeota, and Aenigmatarchaeota. A substantial proportion (3.86%) of sequences remained unclassified (“Unclassified Archaea”), highlighting the presence of unknown or uncultured archaeal diversity. (**b**) Stream graph depicting the proportional distribution of archaeal lineages across individual samples (A–E). Each color band represents a specific archaeal taxon as shown in the legend. The vertical axis denotes the relative abundance (0–100%) of each group within each sample. Samples B and D display the broadest taxonomic diversity, while modern Samples A and E show higher dominance of Halobacteria and reduced representation of deeper clades. These patterns reflect a combination of depositional environments, preservation conditions, and possible microbial community succession during sabkha evolution.

Archaeal community structure across sabkha samples was reconstructed using protein datasets mapped via UniProt accession IDs, followed by phylum-level taxonomic classification (Figure 5a). Major archaeal lineages identified include Euryarchaeota, Crenarchaeota, Thaumarchaeota, Nanoarchaeota, and Bathyarchaeota. As anticipated for a hypersaline ecosystem [63], Euryarchaeota was the dominant phylum, with Halobacteria accounting for 15.53% of annotated proteins (Supplementary Materials S2). However, the overall archaeal diversity was unexpectedly high, with a broad distribution of candidate and uncultured lineages, and approximately 3.86% of proteins assigned to “Unclassified Archaeon,” highlighting both the dynamic taxonomy of Archaea and the current limitations of reference databases.

Thaumarchaeota were consistently detected across samples, contributing 2.86% of total archaeal protein assignments. Their presence is noteworthy given their historical misclassification—some Thaumarchaeota members, particularly ammonia-oxidizing archaea (AOA), were previously annotated as mesophilic marine bacteria [64]. Their detection in hypersaline sediments suggests potential roles in nitrogen cycling within the sabkha system, although specific functional assignments remain limited due to database resolution.

All samples shared a core archaeal structure composed of Euryarchaeota, Crenarchaeota, Candidatus Bathyarchaeota, and Candidatus Woesearchaeota, but sample-specific taxonomic shifts were evident (Figure 5b). Sample B exhibited the widest taxonomic coverage, lacking only Lokiarchaeota and Candidatus Nezhaarchaeota. In contrast, Samples A and E, both modern deposits, showed higher dominance of Halobacteria and reduced representation of deeper-branching lineages. These patterns likely reflect both environmental filtering and differential preservation potential.

Particularly intriguing is the presence of thermophilic lineages such as Thermoplasmata (Euryarchaeota) and Crenarchaeota—typically associated with high-temperature habitats like hydrothermal vents and terrestrial hot springs [65]. Their appearance in a hypersaline, surface-exposed system suggests possible paleoenvironmental thermal inputs, ecological flexibility, or the preservation of ancient protein signatures introduced through sedimentary processes.

While proteomic taxonomic reconstruction lacks the genomic resolution of metagenomics, it remains an essential tool in systems where DNA preservation is poor or compromised. In this context, the taxonomic diversity revealed from protein assignments provides key insights into microbial community dynamics, survival strategies, and the role of environmental and diagenetic factors in shaping the preserved biosignature record.

### 2.4. Early Preservation Windows and the Astrobiological Significance of Poorly Characterized Proteins

Importantly, our results underscore that the mineralogical context of sedimentary deposits exerts a dominant control over protein preservation, with significant implications for biosignature detection on Mars. We observed that carbonate- and silica-rich matrices—especially those dominated by calcite, aragonite, and α-quartz—provided the most favorable conditions for the long-term stabilization of structurally complex proteins, including DNA-binding and metal-coordinating molecules. These environments promote preservation through early mineral entrapment, reduced water activity, and favorable electrostatic interactions between protein residues and mineral surfaces [66]. Such preservation pathways align closely with mineralogical features observed in key Martian terrains, including Jezero Crater, Nili Fossae, and Terra Sirenum, where carbonates and amorphous silica are widespread and often associated with ancient aqueous environments [67,68].

At the same time, while halite- and gypsum-dominated sabkha facies showed lower protein yields in our study, this appears to reflect reduced microbial input rather than poor preservation potential. In fact, halite-rich matrices may offer exceptional molecular stability due to their ability to promote desiccation, exclude oxygen, and suppress enzymatic degradation [33,69]. This dual role—limiting biomass but enhancing preservation—suggests that chloride-bearing terrains on Mars, such as those in the southern highlands, may serve as highly effective molecular “cold traps” for ancient biosignatures [7]. Thus, Martian environments with either carbonate–silica or chloride mineral assemblages represent high-priority targets for future life detection missions. Their complementary taphonomic advantages—carbonate–silica matrices facilitating early stabilization and mineral–protein binding, and chlorides promoting long-term molecular entombment—enhance the likelihood that preserved biomolecules, particularly proteins, may still persist within Martian sedimentary records.

We recognize, however, that the temporal window examined in this study—from the present day to ~11,000 years BP—is relatively short compared to the billion-year timescales relevant for Mars. Yet, our results strongly suggest that the most critical phases of molecular preservation occur during the earliest moments following burial. Early diagenetic mineralization, particularly in carbonate–silica matrices, plays a decisive role in shielding proteins from enzymatic degradation, oxidation, and hydrolysis, enabling long-term stability [70]. Thus, while our dataset does not extend into deep time, it effectively captures the initial conditions that determine whether molecular preservation can occur in the first place—a prerequisite for the survival of biosignatures on planetary timescales.

To strengthen the temporal relevance of biosignature research, future investigations should expand to older analog materials, such as Pleistocene or Miocene evaporites, which offer extended preservation intervals more comparable to Martian timescales. Additionally, controlled degradation experiments under simulated Martian surface and subsurface conditions would help refine our understanding of protein stability over geological time. Such efforts would bridge the current gap between Holocene-scale observations and the billion-year preservation scenarios applicable to Mars [62,69]. Moreover, integrating complementary biosignature detection techniques—including lipidomics, isotopic fractionation, and high-resolution spatial tools such as NanoSIMS or ToF-SIMS—will be essential to constrain molecular residence times and preservation pathways in extreme sedimentary environments.

An additional limitation stems from the fact that approximately 12% of the recovered protein dataset remains unclassified, reflecting both the intrinsic challenges of paleoproteomic analyses and current gaps in extremophile reference databases. This ambiguity highlights a broader issue in microbial taxonomic classification, particularly in environments dominated by poorly characterized or uncultured archaeal lineages [71,72]. In the context of our study, many of these unclassified proteins may originate from halophilic or polyextremophilic organisms with no close representation in existing databases, thereby escaping confident annotation. This lack of taxonomic resolution constrains our ability to fully reconstruct community structure, metabolic capabilities, and ecological succession across the sabkha chronosequence.

To address this, future efforts should prioritize the expansion and curation of extremophile proteomic libraries, particularly those incorporating halophilic, thermophilic, and acidophilic taxa from natural analog environments [73]. Complementary approaches such as metaproteogenomic integration, which combines proteomic data with metagenomic or single-cell genomic frameworks, can also improve annotation confidence by anchoring proteins to broader genomic context [74]. Moreover, leveraging machine learning-based functional prediction tools—including deep learning models trained on structural and biochemical properties—may offer alternative routes for identifying protein roles and lineage affiliations, even in the absence of direct homologs [75]. Ultimately, recognizing and systematically investigating these unclassified proteins may not only improve taxonomic clarity but also uncover novel biochemical adaptations and biosignature targets relevant to life detection on Mars and other planetary bodies.

## 3. Materials and Methods

### 3.1. Geologic Settings

To investigate early molecular preservation in hypersaline deposits, sabkha materials of varying ages were sampled to assess the degree of molecular degradation over the first tens of thousands of years. Sabkhas are coastal, arid environments characterized by flat, salt-encrusted surfaces formed through the evaporation of shallow saline groundwater. As water evaporates, salts and other minerals precipitate, producing a hardened surface crust. Beneath this crust, layers of soft mud or clay often persist, which can support cyanobacterial communities during episodic flooding events. These environments closely resemble ancient saline deposits on Mars, such as those identified in regions like Terra Sirenum [7], which eventually could contain biomolecule remains if life ever arose on Mars.

**Figure 6:**
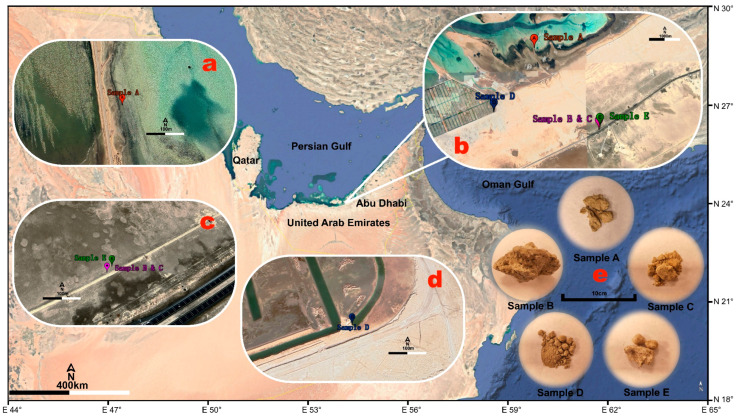
Distribution of sample locations across sabkha units of different ages along the northern coast of the United Arab Emirates. (**a**) Sampling locations at various outcrops, including Sample A from modern deposits and Samples B and C from a young sabkha terrace. (**b**) Detailed view of the sampling site for Sample A in the modern sabkha. (**c**) Image of the lower sabkha terrace where Samples B and C were collected, along with the location of Sample E, which was obtained from a precipitated brine in modern sabkha waters. (**d**) Upper sabkha terrace showing the sampling site for Sample D. (**e**) Overview of the five analyzed samples used for protein studies.

The Abu Abyad sabkha system, situated along the western coast of Abu Dhabi, United Arab Emirates, represents one of the most extensive modern coastal sabkha environments in the world (Figure 6) [14]. The region has developed within the Arabian Gulf’s low-angle carbonate ramp setting, where the interaction between marine processes and the arid climate has created ideal conditions for sabkha formation [76]. The coastal plain of Abu Dhabi is characterized by a complex mosaic of sedimentary environments, including intertidal flats, microbial mats, and evaporitic deposits that have evolved throughout the Holocene Period [77].

In this sedimentary context, the sample collected in the modern mudflat environment (24.146241° N, 54.083526° E) represents the most recent phase of sabkha development, featuring active cyanobacterial mat communities within fine-grained carbonate and siliciclastic sediments (Figure 6a,b). These modern deposits are subject to regular tidal influences and seasonal fluctuations in groundwater levels, creating optimal conditions for microbial mat formation and early diagenetic mineral precipitation [78,79]. The distinctive laminated structure of these deposits results from the cyclic growth of microbial mats and subsequent sediment trapping, binding, and early mineralization processes [80].

The chronostratigraphy of the Abu Dhabi sabkha system reveals distinct depositional phases tied to Quaternary sea-level fluctuations. The mid-Holocene sabkha terrace (24.101798° N, 54.123786° E) represents deposits formed approximately 5000–7000 years ago during the well-documented mid-Holocene highstand, when sea levels were 1–2 m above present levels [81,82]. This terrace preserves evidence of complete sabkha development cycles (Figure 6a,c) and exhibits advanced stages of evaporite mineral precipitation and diagenetic alteration, characterized by distinctive polygonal surface patterns and multiple generations of gypsum and anhydrite formation. These features reflect the complex interplay between carbonate precipitation and evaporite formation that occurred under the influence of highly saline groundwater during its active development phase [50].

The Early Holocene sabkha deposits (24.111436° N, 54.057219° E) correspond to the initial marine transgression in the Arabian Gulf following the Last Glacial Maximum (Figure 6a,d). Radiocarbon dating places these deposits at approximately 9000–11,000 years ago, when rapid deglacial sea-level rise first flooded the Gulf basin [81,82]. These deposits contain well-preserved sedimentary structures and complex mineral assemblages, including dolomite, gypsum, and anhydrite, providing crucial information about environmental conditions during this early transgressive phase of the Holocene.

The most ancient sabkha terrace in the study area represents preserved Pleistocene sequences formed during the last interglacial period. Optically stimulated luminescence (OSL) dating confirms these elevated deposits were formed during Marine Isotope Stage (MIS) 5e, approximately 120,000–130,000 years ago, when sea levels were approximately 6–8 m higher than present [83,84]. The preservation of biological materials within these ancient deposits, coupled with their diagenetic histories, offers valuable insights into long-term molecular taphonomy under hypersaline conditions.

This natural chronosequence, encompassing modern, Holocene, and Pleistocene sabkha deposits, provides an exceptional opportunity to study the evolution of coastal evaporitic systems and their response to environmental change over geological time scales. Understanding these processes is particularly relevant for interpreting ancient sabkha deposits worldwide and for predicting the potential impacts of future climate change on coastal environments [85].

### 3.2. Site Description and Sampling Procedures

To investigate the mechanisms of early protein degradation and preservation processes in sabkha environments, systematic sampling was conducted across three distinct sabkha units within the Abu Abyad region, United Arab Emirates (Figure 6). The sampling strategy was designed to capture a temporal sequence of sabkha development, from modern to ancient deposits.

The first sampling site (Sample A; 24.146241° N, 54.083526° E) represents an active mudflat environment characterized by well-developed cyanobacterial mats, providing baseline data for initial protein compositions in modern sabkha systems. The second sampling location yielded two distinct sample sets (Samples B and C; 24.101798° N, 54.123786° E) from a younger terrace, representing an intermediate stage of sabkha development. The third site (Sample D; 24.111436° N, 54.057219° E) comprised materials from an older sabkha terrace, allowing examination of long-term protein preservation patterns.

Rigorous sampling protocols were implemented to minimize contamination during sample collection. Sediment samples were obtained from freshly exposed internal sections of outcrops after removing the weathered surface material. All samples were collected using sterilized stainless-steel tools, with personnel wearing nitrile gloves throughout the sampling process. Immediately after collection, samples were sealed in sterile polyethylene bags, labeled, and stored under controlled conditions to maintain sample integrity. This standardized sampling procedure was consistently applied across all sites to ensure sample quality and analytical reproducibility.

### 3.3. Radiocarbon Dating (14C)

Radiocarbon dating analyses were performed at Beta Analytic Laboratory (Miami, Florida, USA). While Samples A and E represent modern deposits serving as reference materials, and Sample C was collected from a stratigraphically equivalent horizon to Sample B, only Samples B and D were selected for age determination. Following Beta Analytica’s standard protocols, 2-g aliquots of Samples B and D were prepared by gentle desiccation at 30 °C for 24 h prior to shipping to the laboratory for radiocarbon analysis.

### 3.4. Mineral Identification by X-Ray Diffraction (XRD)

The mineral identification of the Samples A to E was done through the X-ray diffraction technique. For such a purpose, a sample portion was powdered using an agate mortar. The mineral characterization was done by X-ray diffractometer (SmartLab SE, Rigaku, Tokyo, Japan) using Cu-Kβ radiation, the sample powder was scanned in 2θ° diffraction angles from 5 to 80° at a step of 0.01° and a count time of 1 s per step.

### 3.5. Protein Extraction and Concentration

Prior to protein extraction, samples were powdered using an agate mortar and pestle that had been sterilized and sequentially cleaned three times with analytical-grade methanol. For each sample, 10 g of powdered material was accurately weighed using an analytical balance. Protein extraction was performed using a mass spectrometry-grade Metaproteome Extraction Kit (Bangfei Company, Beijing, China) following the manufacturer’s protocol (detailed in Supplementary Materials S1), yielding 400 μL of protein solution per sample.

Protein quantification was conducted using a fluorescence-based assay with a Qubit 4 Fluorometer (Thermo Scientific, Waltham, MA, USA) and the Qubit™ Protein BR Assay Kit (ThermoFisher, Waltham, MA, USA). The assay was performed in duplicate for each sample and standard. The reaction mixture consisted of 20 μL of sample or standard, 150 μL of Qubit Protein BR Assay Buffer, and 30 μL of Qubit Protein BR Assay Reagent. Following reagent addition, tubes were immediately vortexed for 5–7 s and incubated at room temperature for 10 min before measurement on the Qubit 4 Fluorometer. Each extracted protein sample was analyzed in triplicate to ensure measurement accuracy (Supplementary Materials S1).

### 3.6. Trypsin Digestion and Desalting

To prepare proteins for mass spectrometry analysis, samples were subjected to reduction, alkylation, and enzymatic digestion. Trypsin was selected as the proteolytic enzyme due to its high specificity for cleaving peptide bonds at the carboxyl side of lysine and arginine residues, generating peptides of suitable length and charge state for optimal mass spectrometric analysis [86]. The protein samples were first diluted with an equal volume of 50 mM ammonium bicarbonate buffer. Disulfide bonds were reduced by adding 200 mM dithiothreitol (DTT) to a final concentration of 10 mM, followed by incubation in a water bath at 60 °C for 30 min. Subsequently, protein alkylation was performed by adding 100 mM 2-iodoacetamide (IAM) to a final concentration of 10 mM to prevent disulfide bond reformation. Enzymatic digestion was initiated by adding 20 μL of trypsin (stored at −80 °C) and incubating the samples overnight (minimum 16 h) in a 37 °C water bath.

Following digestion, the reaction was terminated by adding 1 μL of formic acid. The resulting peptide mixture was desalted using Zeba™ Spin Desalting Columns (7K MWCO, 2 mL, ThermoFisher Scientific Waltham, MA, USA). The desalting procedure began with column preparation by removing the bottom closure and loosening the cap. The column was placed in a collection tube and centrifuged at 1000× *g* for 2 min to remove storage solution. A mark was placed on the side where the compacted resin was slanted upward to maintain proper column orientation during subsequent centrifugation steps.

Column equilibration was performed by adding 1 mL of buffer or ultrapure water, followed by centrifugation at 1000× *g* for 2 min. This washing step was repeated two to three times, with the flow-through being discarded after each step. For sample processing, the column was transferred to a new collection tube, and the sample was applied slowly to the center of the resin bed. For low-volume samples, a layer of ultrapure water or buffer was added after sample absorption to ensure maximum peptide recovery. The samples were then centrifuged at 1000× *g* for 2 min, and the desalted peptides were collected for subsequent mass spectrometry analysis.

### 3.7. LC-MS Analysis

Liquid chromatography-mass spectrometry (LC-MS/MS) analysis was performed at Bangfei Biotechnology Co., Ltd (Beijing, China). using a Thermo Scientific Orbitrap Astral mass spectrometer coupled to a Vanquish Neo uHPLC system equipped with a µPAC Neo HPLC column (Thermo Scientific, Waltham, MA, USA). The mobile phases consisted of Buffer A (0.1% formic acid in water) and Buffer B (0.1% formic acid in 80% acetonitrile). Prior to analysis, the chromatographic column was equilibrated with 100% Buffer A.

Samples were automatically loaded onto a mass spectrometry pre-column before separation on the analytical column. Chromatographic separation was performed at 60 °C using a gradient elution program at a constant flow rate of 2.5 μL/min. The gradient profile was as follows: initial conditions of 4% Buffer B; 20% Buffer B at 4 min; 35% Buffer B at 5.8 min; 99% Buffer B at 6.2 min, maintained until 7 min. Detailed mass spectrometric parameters are provided in Table 1.

The mass spectrometry proteomics data have been deposited to the ProteomeXchange Consortium (accessed on 13 January 2025, http://proteomecentral.proteomexchange.org) via the iProX partner repository [87,88] with the dataset identifier PXD058693. The analytical parameters used in this study are detailed in Table S1 of the Supplementary Materials S1.

### 3.8. Direct DIA (Data-Independent Acquisition) Database Searching

Mass spectrometry data files generated by the Orbitrap Astral mass spectrometer were acquired in Raw format and analyzed using MaxDIA suite (MaxQuant version 2.6.2.0) for direct data-independent acquisition (DIA) analysis. This library-free approach employs deep learning algorithms to generate in silico spectral libraries by predicting fragment ion intensities and retention times for peptide sequences from the complete proteome or target protein families [89].

For protein identification, searches were performed against the complete archaeal protein sequence dataset downloaded from the UniProtKB database (accessed on 10 July 2024, https://www.uniprot.org/uniprotkb). The MaxQuant contaminant database was included to identify potential contaminating proteins. Search parameters were configured as follows: enzyme specificity was set to trypsin with C-terminal cleavage after lysine and arginine residues allowing for proline restriction (Trypsin/P); variable modifications included methionine oxidation and N-terminal protein acetylation, while carbamidomethylation of cysteine was set as a fixed modification. The “Up to first space” identification rule was applied, and label-free quantification was enabled using the MaxQuant LFQ algorithm.

To ensure high confidence in protein identification, both peptide-spectrum match false discovery rate (PSM FDR) and protein false discovery rate (Protein FDR) were stringently set to 1% (0.01). Default settings were maintained for all remaining parameters in the Group-specific parameters tab and MS/MS analyzer settings. The resulting peptide and proteome files were subsequently analyzed for protein identification and quantification.

## 4. Conclusions

This study examined the preservation of proteins in modern and ancient sabkha environments in Abu Dhabi as a terrestrial analog for the potential retention of molecular biosignatures on Mars. Using proteomic profiling across a Holocene chronosequence, we identified 722 protein groups and 1300 peptides, uncovering a complex interplay between environmental conditions, microbial community composition, and mineralogical context that governs long-term protein preservation.

We observed that carbonate-rich matrices, particularly those enriched in calcite and silica, were most conducive to long-term preservation, supporting the survival of both metal-binding proteins and positively charged DNA-binding proteins via mineral–protein interactions. In contrast, halite- and gypsum-dominated samples preserved fewer proteins, likely due to both reduced original biomass and the chemically aggressive nature of these evaporitic minerals. Protein degradation patterns were not linear with time; modern samples (A and E) yielded predominantly shorter proteins, while intermediate-aged samples (B and C) preserved larger, multi-domain proteins—such as Gingipain R and UbiD family decarboxylases—suggesting that early encapsulation and favorable mineral matrices outweigh mere chronological age in determining preservation.

The preserved protein profile reveals a profound narrative of microbial physiological adaptation to extreme environmental challenges, where metabolic enzymes, environmental response regulators, and DNA-associated proteins dominate the functional landscape, demonstrating how microorganisms actively reconfigure their proteome in response to harsh conditions. In older samples, a striking shift emerges towards “genome maintenance” functions, with an increased presence of DNA repair enzymes and ribosomal proteins, reflecting the microorganisms’ immediate physiological strategies for survival, not as a predictive mechanism, but as a real-time cellular response to prolonged nutrient limitation and hypersalinity. Critically, these ancient proteins were most likely preserved during the initial moments of environmental interaction—specifically during early mineralization phases—with Sample D’s rapid carbonate–silica entombment capturing the precise proteomic signature of stressed microbial communities at the exact moment of environmental challenge. This preservation method effectively “freezes” the microorganisms’ instantaneous physiological state, providing an unprecedented view of their immediate adaptive responses and serving as a molecular testament to microbial resilience, showcasing how life dynamically reconfigures its molecular machinery to persist in extreme conditions, moment by moment, without anticipating future challenges but responding directly to current environmental pressures.

Taxonomically, proteomics revealed unexpected archaeal diversity, with broad representation of lineages such as Euryarchaeota, Crenarchaeota, Bathyarchaeota, and Thaumarchaeota. The latter’s presence, alongside thermophilic taxa like Thermoplasmata, suggests either environmental flexibility or ancient thermal imprints in the sedimentary record. While proteomic taxonomic resolution remains limited compared to DNA-based methods, our findings demonstrate that protein data can reconstruct microbial community structure in extreme environments where DNA degradation is advanced.

Importantly, our results underscore a shift in preservation mode over time, from physical encapsulation in modern sabkha deposits to chemical stabilization in ancient ones, mediated by mineral–protein interactions and microenvironmental buffering. This shift has profound implications for Mars exploration: it suggests that biosignatures on Mars, if present, will likely be biased toward specific protein classes with structural or chemical features favoring preservation. Consequently, carbonate- and silica-rich evaporitic facies on Mars, particularly those formed under moderate salinity and redox conditions, should be prioritized in the search for ancient molecular remnants.

Ultimately, our findings support the use of sabkha systems as robust analogs for Mars and demonstrate that even under extreme conditions, preserved proteins can retain taxonomic, ecological, and functional information over millennial timescales—an encouraging sign for astrobiological exploration.

While this study provides new insights into protein preservation in sabkha environments, several limitations remain. The temporal window examined—present day to ~11,000 years BP—is short compared to the billion-year timescales relevant for Mars, making extrapolation inherently uncertain. Additionally, despite careful protocols, the contribution of modern microbial input to ancient protein profiles cannot be fully excluded.

Proteomic identification also faces taxonomic ambiguity due to protein homology and the limited resolution of existing databases for extremophiles. Expanding reference datasets and developing tailored spectral libraries for ancient and halophilic proteins will be essential to improve future analyses.

Looking ahead, experimental degradation studies under Mars-analog conditions, analysis of older evaporitic sequences, and integration with complementary biosignature tools (e.g., lipids, isotopes, NanoSIMS) will help refine our understanding of long-term molecular preservation. These efforts will strengthen our ability to identify robust biosignatures in extreme environments—on Earth and beyond.

## Figures and Tables

**Table 1:** Results from the sabkha samples, describing their protein content, estimated age, and mineral composition. Total protein content (TPC) is given in ng-μL^−1^, along with protein and peptide characteristics. The Prot/pep ratio represents the proportion of proteins to peptides, while the length range indicates the observed protein size distribution. Average age (^14^C) is provided where available. Mineral composition and estimated salinity (g-L^−1^) reflect the geochemical conditions of each sample.

Sample	TPC	Proteins	Peptides	Prot/Pep Rate	Length Range	Average Age Yr (^14^C)	Mineral Composition	Estimated Salinity
Sample A	540	125	244	0.51	79–1420	Modern Sediment	Aragonite/Calcite	40–70
Sample B	438	405	539	0.75	92–964	2918–2502 Cal BP	Aragonite/Calcite/Mg Calcite	60–90
Sample C	452	48	164	0.29	78–3876		Gypsum/Halite	100–150
Sample D	494	97	213	0.46	86–1349	11,231–10,770 Cal BP	Calcite/SiO_2_	50–100
Sample E	332	47	140	0.34	96–1975	Modern Sediment	Halite	300–350

## Data Availability

Data is contained within the article and Supplementary Material.

## Supplementary Materials

The supporting information can be downloaded at: https://www.mdpi.com/article/10.3390/ijms26135978/s1.
