# Supplementary material for: Comparing Protein Stability in Modern and Ancient Sabkha Environments: Implications for Molecular Remnants on Ancient Mars
Source: Int J Mol Sci. 2025 Jun 21;26(13):5978. doi: 10.3390/ijms26135978 (PMC12250420; doi:10.3390/ijms26135978)
Supplement: Supplementary file 1 [file ijms-26-05978-s001.zip › ijms-3597639_Supplementary Materials S1_rev2.pdf]

Supplementary Material

Comparing protein stability in modern and ancient sabkha environments: implications for molecular remnants on ancient Mars

Qitao Hu[1,2], Ting Huang[1,2], Aili Zhu[1,2], Angelica Angles[3], Osman Abdelghany[4], Ahmed Aal[4], David C. Fernández-Remolar[1,2,5,*]

[1] State Key Laboratory of Lunar and Planetary Sciences, Macau University of Science and Technology, Macau 999078, PR China
[2] CNSA Macau Center for Space Exploration and Science, Macau 999078, PR China
[3] Geosciences department, College of Sciences, United Arab Emirates University, Al Ain, UAE
[4] Blue Marble Space Institute of Science, Seattle, WA, United States
[5] ISA, Iceland Space Agency, 101 Reykjavik, Iceland
* Correspondence should be addressed to D.C.F.-R. (email: dcfremolar@must.edu.mo)

1. Methodology: Supplementary Protocols for Protein Extraction and Quantification
2. Table S1

Supplementary Protocols for Protein Extraction and Quantification

1. Protein Extraction Protocol from Banfei

1.1 Sample Preparation and Initial Extraction

Approximately 5–10 g of each sample was transferred into a 50 mL centrifuge tube, and 15–30 mL of Reagent A was added. Tubes were vortexed at 3000 rpm for 15 min to ensure complete homogenization.

1.2 Protein Solubilization

An equal volume of Reagent B was added to each tube. After thorough mixing, samples were incubated at 4 °C (or on ice) for 30 min and subsequently centrifuged at 15,000×g for 15 min at 4 °C.

1.3 First Re-Extraction

The supernatant was carefully transferred to a fresh centrifuge tube. An equal volume of Reagent A was added, followed by vortexing to achieve full mixing. Samples were centrifuged again at 15,000×g for 15 min at 4 °C.

1.4 Repeat Extraction

Steps 1.2–1.3 were repeated once to maximize protein recovery.

1.5 Protein Precipitation

The resulting supernatant was transferred to a new centrifuge tube, and five volumes of Reagent C were added. Samples were incubated at −20 °C for a minimum of 2 h or overnight to precipitate proteins.

1.6 Protein Pellet Recovery

Samples were centrifuged at 15,000×g for 15 min at 4 °C, and the supernatant was discarded. The resulting pellet was retained.

1.7 Methanol Wash

To wash the pellet, 1 mL of pre-cooled methanol was added. The pellet was resuspended gently by pipetting.

1.8 Methanol Centrifugation

Samples were centrifuged at 15,000×g for 5 min at 4 °C. The supernatant was discarded.

1.9 Acetone Wash

The pellet was resuspended in 2 mL of pre-cooled acetone by gentle pipetting.

1.10 Acetone Centrifugation

Centrifugation was carried out at 12,000 rpm for 5 min at 4 °C, and the supernatant was discarded. Steps 1.9–1.10 were repeated once to ensure thorough washing.

1.11 Drying and Solubilization

The final pellet was air-dried at room temperature, then dissolved in an appropriate volume of Reagent D. The suspension was incubated overnight at 4 °C or for 2 h at 22 °C to ensure complete solubilization.

1.12 Final Clarification

The samples were centrifuged at 15,000×g for 15 min at 20 °C. The resulting supernatant, containing the purified protein extract, was either used immediately for downstream analysis or stored at −80 °C for future use.

2. Protein Concentration Determination

2.1 Preparation of Standards and Samples

I. Ten 0.5 mL microcentrifuge tubes were labeled for standards, blanks, and samples, including:

II. One tube each for Standard 1 and Buffer Blank

III. Five tubes for Sabkha samples

IV. Three replicate tubes for Standard 2, to improve calibration reproducibility

V. 20 μL of each Qubit™ Protein BR Standard (Standard 1 and 2) was added to the designated tubes, followed by 150 μL of Qubit™ Protein BR Assay Buffer. Samples were mixed by pipette

VI. 20 μL of each protein sample was pipetted into corresponding tubes. 150 μL of Qubit™ Protein BR Assay Buffer was added to each, and the mixtures were pipetted up and down to mix. Final volume: 170 μL.

VII. 20 μL of buffer was added to the buffer blank tube, followed by 150 μL of Qubit™ Protein BR Assay Buffer. Samples were mixed thoroughly.

VIII. 30 μL of Qubit™ Protein BR Assay Reagent was added to each tube (samples, standards, and buffer blank), followed by immediate mixing by vortexing for 5–7 seconds. Final volume of each tube: 200 μL.

IX. Tubes were incubated at room temperature for 10 min before fluorometric measurement.

2.2 Fluorometric Quantification

I. On the Qubit™ 4 Fluorometer, select Protein > Protein Broad Range from the home screen.

II. Press Read Standard, then insert the tube with Standard 1, close the lid, and press Read Standard. Remove the tube after reading (~3 seconds).
3. Insert each Standard 2 replicate, read them consecutively, and use the average RFU values for calibration.

III. Once all standards are read, press Run Samples.

IV. Set sample volume to 20 μL and units to ng/μL on the assay screen.

V. Insert the Sample Buffer Blank tube and press Read Tube.

VI. Insert each sample tube, one by one, and press Read Tube. Results are displayed on the screen.

VII. Repeat step 7 for all protein extracts. Note: Each sample was measured in triplicate to ensure accuracy and reproducibility.

Table S1. Analytic parameters with Orbitrap Astral mass spectrometer.

**Main analytic parameters of full MS**	
Runtime (min)	7
Polarity	Positive
MS main features	
Resolution	240000
AGC target (%)	500
Maximum Injection Time (ms)	5
Scan Range (m/z)	380-980
Main DIA (data independent acquisition) parameters	
Runtime (min)	7
Polarity	Positive
Default charge state	2
Advanced Peak Determination	Ture
MS2	
Resolution	80000
Precursor Mass Range (m/z)	380-980
DIA Window Type	Auto
Isolation Window (m/z)	2
Number Of Scan Events	299
DIA Window Mode	m/z Range
Collision Energy Type	Normalized
HCD Collison Energy (%)	25
Detector Type	Astral
Scan Range (m/z)	150-2000
AGC target (%)	500
Maximum Injection Time (ms)	3
Microscans	1
Loop Control	Time
Time(sec)	0.6
